# Can We Use Commercial Mobile Apps Instead of Research Mobile Apps in Healthcare Research?

**DOI:** 10.3389/fpubh.2021.685439

**Published:** 2021-07-23

**Authors:** Emre Sezgin

**Affiliations:** The Abigail Wexner Research Institute at Nationwide Children's Hospital, Columbus, OH, United States

**Keywords:** mobile health, mobile app, mHealth, research app, healthcare research

## Introduction

Scientific research has been at the front line of unlocking many innovations and opportunities as well as creating the evidence for methods and technologies. Similarly, there has been an increasing effort on mobile health research to tackle healthcare communication and access problems at individual and population level. However, there have been a number of problems limiting the progress of mobile health research. The major road blocker has been the sustainability and reach of RMA in real world use. This created a disparity between academia and industry in the development of digital health solutions and improving health outcomes for populations (1). Research for developing apps and creating evidence take time and heavily depend on research fund within pre-determined project period. Eventually, completing a research app prototype might take years. Whereas, the industry has been on the fast track in development, as creating sustainable pipeline for emerging products and apps. Inevitable systemic differences have been mostly the reason to apart the path of these two domains.

Even though there has been occasional high-level convergence between industry and academia (e.g., Apple Heart Study with Stanford U) (2), the disparity mostly resulted as an increase in digital waste and lack in long-term improvements in digital health and impact on health outcomes. This problem has not been one-sided, it also affected CMA adversely, as most of the academic findings remained underutilized by the industry. Earlier studies showed that many of commercial health apps were not sufficiently generating evidence or using the science in design, which impact the utility of their apps and health outcomes (3, 4).

The symbiotic relationship of RMA and CMA is apparently necessary regarding the benefits for research and industry. So, the question is: “How could we utilize CMA in healthcare research through sustainable and scalable apps that can inform the science more effectively?”

## Converging Commercial Mobile APP and Research Mobile APP

CMA have had the upper hand with their access to a large scale of users, exposure and awareness, and associating the app with current needs (e.g., Insurance apps to check policy, driving score, submitting claims, and insurance card details). However, it is tricky to use an app on the market for research. First, the lifecycle of any new app, especially in mobile health, is unpredictable. There is no guarantee that the app will remain on the market throughout the research period or later. This makes the apps in the market less desirable for a researcher to collaborate. Second, it is hard for a researcher to get the buy-in from developers of existing apps in the market to support research in their existing product line. Legal and operational requirements (including privacy, security, and functionality requirements) between institutions to share data and to do modifications in CMA may not be feasible for both sides. On the other side, academia may not be a desirable business partner due to slow progress in research, potentially short-lasting business relationships and low return on investments (depending on the research grant). As research requires rigorous and steady steps and preferably low-risk methods, industry can accommodate taking risk and adopt fail fast and recover approach. Eventually, the different dynamics of these two domains lead researchers to develop a research app instead of using an app in the market.

Relatively, RMA development timeline is shorter than CMA, mostly within a year or less depending on the project and funding mechanism. The immediate next step after development is finding or “recruiting” the users. Similarly, that process is within a short timeline. These tight timelines limit the ability to develop comprehensive and scalable RMA, and also to establish user basis and awareness for an app. Eventually, regardless of targeted populations, retention rate on RMA use is low due to the fact that majority of the studies are incentive-based, and RMA is supported for limited time. Therefore, the end users or participants drop using RMA once study is over, get paid and RMA is removed from app stores. Short lifecycle of RMA adversely impacts scientifically valuable observational data, e.g., long-term digital impact and observation of behavior change. Whereas, CMA can enable long-term scientific observations as well as being promising in effective intervention design (5). Very few researchers were successful to conduct long term observations through RMA, if they secure a continuing funding or commercialization pathway.

A fundamental piece of mobile apps is the integration of core values such as user characteristics, daily needs, and lifestyle. Considering personal and environmental variables, such as socioeconomic status, technology literacy and adoption, and personal lifestyle and preferences, there are multiple factors that could affect a person's decision toward using a specific technology. Behavior change techniques (BCT) and digital behavior change interventions (DBCI) have been used to investigate promotion of healthy behavior using RMA (6). However, these methods do not provide an optimal solution for a sustainable research app strategy. The question is “What would be the cost-effective and sustainable method for mobile-based health interventions?” To find the answer, most of the CMA are spending a lot of time in market research, investing in infrastructure, funded by long-term investments, and have teams for long-term commitment and advertising efforts. For a research app, these may not be viable options, but this should not stop a researcher learning from business strategies of CMA and leverage them to converge with research outcomes.

## Need for Using the “Defaults”

As behavioral economics principles suggest, avoiding complex decisions and potential learning curve barrier, using defaults to nudge toward the right direction with existing methods would potentially yield higher returns (7). Applying this phenomenon in using default on mobile apps, a default commercial mobile app (dCMA) based research and intervention is necessary. dCMA is a subset of CMA which refers to default apps on the phones or commonly used apps by many, such as, notes, navigation, calendar, and reminder apps, YouTube and Google apps (Android phones), iTunes, FaceTime, Screen Time, and Health apps (Apple iPhones). Given the shortcomings of a CMA (e.g., reliability and sustainability issues from a research perspective), dCMA approach could be scalable, interoperable and cost-effective regarding mainstream development, design, and training efforts for an app. dCMA has a potential for higher retention rates by leveraging existing adopted use and lower cognitive workload. Actually, text messages had been one of the early utilizations of dCMA, and text message-based interventions have been a core for mobile health research for a long time. Because SMS is easy and user-aware method, and phone numbers enable persistent connection with the user on a widely adopted self-sustaining platform. However, it has limitations in terms of interactive multimedia communications and rich feedback through sensors. Eventually, it is inevitable to shift the research efforts on mobile platforms utilizing multimedia tools or multimodal approaches.

An extension of dCMA is the use of on-the-phone alarms or calendars for medical appointment reminders. Why cannot we use a default reminder app (dCMA) instead of building an additional app (RMA) for it in research? The use of dCMA over RMA or any other CMA has also been observed among providers, while providers use default chat apps instead of dedicated apps for clinical discussion (8). Using dCMA inherently have a higher adoption in medical adherence (e.g., having the habit of using reminders for medicine) (9). But one major obstacle with default apps is the remote access and data collection in research. Not being able to control the app and acquire data from commercial companies, researchers would need users to share data continuously throughout a research. To minimize the dependency in user involvement, new data collection strategies would be required while promoting dCMA use. Sharing screenshots or recordings of mobile apps with researchers is a one of the workarounds to get objective data without depending on commercial companies (10), but it requires high user involvement in data collection which may fail in the long term. A less user-involving approach could be a “ghost” app which can be running in the background without the need of user interaction. Integrated with the other apps that are required for observation, a ghost app can collect data from multiple resources (e.g., location from GPS, activity tracking through Fitness apps) with minimum user involvement. Yet, a ghost app could create a security and privacy concern if not compliant with end-user agreements and could be blocked from accessing data if it is not compliant with operating system (OS) protocols.

Researchers may also leverage dCMA with OS walkarounds, such as through taking part in shared user accounts with users (e.g., using a research Google account or being added to family accounts) which let researchers observe the use of apps through the web portals. This would limit the observation to specific apps and not allow to create interventions (e.g., Search history, YouTube history, browsing history, voice recordings, location history, photos), but could provide vast amounts of insight about user digital behavior for long-term without depending on a CMA or RMA.

Currently, open-source platforms (e.g., Apple ResearchKit and Android ResearchStack) created a gateway to develop integrated data collection apps through APIs, leveraging OS-provided default platforms to reach large number of cohorts (11). It is one step closer to utilize defaults, as Apple enable access to the data collected from their default Health app and Fitness app through ResearchKit and HealthKit (12). It helped to conduct a scaled-up longitudinal study and learn about population health (13). Similarly, RedCAP (myCap app) and FDA (myStudies app) provided white-labeled apps for researchers to leverage APIs and these open-source platforms collecting data through mobile phones in a research (14, 15). Such efforts could advance the partnership among industry and academia toward unifying their paths, and also nurture to be all-inclusive in research regarding the health equity and disparity problem in our society (16, 17).

## Envisioning the Future

In the long term, efforts toward adapting dCMA or CMA more effectively through streamlined independent research platforms could contribute to the knowledge on digital health interventions and behavior research toward improving health outcomes for populations. Further, the knowledge could inform policies and regulations toward improving healthcare and creating guidelines or regulations for adapting and using cross-platform app use and development. Eventually, integrating healthcare research into commercial platforms could be the preferable solution in terms of low cost- high yield longitudinal behavioral and intervention research.

In the ideal world, one possible example is, a platform could exist for researchers to participate, design study and intervention, select the apps to use, identify target population, recruit, and receive consents over the preferred CMA, and do long term observation through a mobile ecosystem. A researcher account could be used for observing target behaviors (e.g., screen time, calls, messaging, social media engagement), prescribing reminders (e.g., Google calendar) for appointments and medication, retrieving physical activity from wearables (e.g., Fitbit) or phone apps using accelerometer sensor, getting mobility behavior through an insurance app, exploring depression or anxiety symptoms *via* triangulating observations from messaging, social media, voice assistant interactions, location, and screen time apps. The inclusion of commercial apps in health would be easily scalable to target populations, easy-to-use and low-cost maintenance and training.

However, the major concerns are revolving around data privacy, trust, data use policies, security, and compliance services. Even though there are significant attempts for privacy and data use policies (e.g., GDPR in Europe), the implementation of prior suggestions needs further exploration on how to leverage commercial apps in healthcare use from regulation, policy, and research perspectives. In addition, necessary steps should be taken to integrate research into a platform, establish multi-institutional agreements, address how to mitigate overhead and additional costs from operational and legal investments for commercial companies, plan return on investment (ROI) for research and industry, and define a roadmap for data requirements, sharing, as well as data ownership and stewardship roles to successfully maintain the research.

## Author Contributions

ES planned and designed the study, completed the research, and wrote the manuscript.

## Conflict of Interest

The author declares that the research was conducted in the absence of any commercial or financial relationships that could be construed as a potential conflict of interest.

## Publisher's Note

All claims expressed in this article are solely those of the authors and do not necessarily represent those of their affiliated organizations, or those of the publisher, the editors and the reviewers. Any product that may be evaluated in this article, or claim that may be made by its manufacturer, is not guaranteed or endorsed by the publisher.
